# Tuberculous meningitis diagnosis and outcomes during the Xpert MTB/Rif era: a 6.5-year cohort study in Uganda

**DOI:** 10.12688/wellcomeopenres.14610.2

**Published:** 2018-07-03

**Authors:** Fiona V. Cresswell, Ananta S. Bangdiwala, Nathan C. Bahr, Emily Trautner, Edwin Nuwagira, Jayne Ellis, Radha Rajasingham, Joshua Rhein, Darlisha A. Williams, Conrad Muzoora, Alison M. Elliott, David B. Meya, David R. Boulware

**Affiliations:** 1 Department of Clinical Research, London School of Hygiene and Tropical Medicine, London, WC1E 7HT, UK; 2 Infectious Diseases Institute, Kampala, Uganda; 3 Division Biostatistics, School of Public Health, University of Minnesota, Minneapolis, MN, 55455, USA; 4 Division of Infectious Diseases, Department of Medicine, University of Kansas, Kansas City, KS, 66160, USA; 5 University of Utah, Salt Lake City, UT, 84112, USA; 6 Mbarara University of Science and Technology, Mbarara, Uganda; 7 Doctors.net.uk, Abingdon, OX14 4SH, UK; 8 Division of Infectious Disease and International Medicine, Department of Medicine, University of Minnesota, Minneapolis, MN, 55455, USA; 9 Medical Research Council/Uganda Virus Research Institute and London School of Hygiene & Tropical Medicine Uganda Research Unit on AIDS, Entebbe, Uganda; 10 College of Health Sciences, Makerere University, Kampala, Uganda

**Keywords:** Tuberculous meningitis, TBM, HIV, diagnosis, outcomes

## Abstract

**Background:** Tuberculous meningitis (TBM), a leading cause of meningitis in sub-Saharan Africa, is notoriously difficult to diagnose. In our Ugandan setting TB diagnostics have evolved rapidly in recent years, with introduction of Xpert MTB/Rif (Xpert) in 2011 and culture in 2013. We aim to describe the impact of improved TBM diagnostics at two Ugandan hospitals between 2010 and 2017.

**Methods: **Adults presenting with meningitis (headache and objective meningism) were assessed for eligibility for enrolment in two consecutive trials investigating cryptococcal meningitis. Cohort one received cerebrospinal fluid (CSF) smear microscopy only (2010-2013). Cohort two received smear microscopy and Xpert on 1ml unprocessed CSF at physician discretion (2011-2013). Cohort three received smear microscopy, routine liquid-media culture and Xpert on large volume CSF (2013-2017) for all meningitis suspects with a negative CSF cryptococcal antigen (crAg). In a post-hoc analysis of three prospective cohorts, we compare rates of microbiologically confirmed TBM and hospital outcomes over time.

**Results: **1672 predominantly HIV-infected adults underwent lumbar puncture, of which 33% (558/1672) had negative CSF crAg and 12% (195/1672) were treated for TBM. Over the study period, microbiological confirmation of TBM increased from 3% to 41% (P<0.01) and there was a decline in in-hospital mortality from 57% to 41% (P=0.27). Adjusting for definite TBM and antiretroviral therapy, and using imputed data, the odds of dying were nearly twice as high in cohort one (adjusted odds ratio 1.7, 95% CI 0.7 to 4.4) compared to cohort three.  Sensitivity of Xpert was 63% (38/60) and culture was 65% (39/60) against a composite reference standard.

**Conclusions: **Since 2010, as TBM diagnostics have evolved, microbiologically-confirmed TBM diagnoses have increased significantly. There has been a non-significant decline in TBM in-hospital mortality but due to multiple possible confounding factors it is not possible to conclude what has driven this decline in mortality.

## Introduction

 Tuberculous meningitis (TBM) is the second most common cause of adult meningitis in sub-Saharan Africa ^1, 2^, accounting for one to five percent of the 10.4 million tuberculosis (TB) cases reported worldwide in 2016 ^3^. Despite treatment, TBM outcomes are poor with 19–28% mortality in HIV-uninfected persons and 40–67% mortality in HIV-infected patients in addition to long-term disability is frequent among survivors ^4– 6^.

Insidious symptom onset in persons with TBM leads to delay in seeking care and increasing disease severity at presentation correlates with higher mortality ^7^. Further, the paucibacillary nature of TBM increases the difficulty in confirming diagnosis once care is sought, also contributing to high mortality ^8^. Cerebrospinal fluid (CSF) smear microscopy for acid-fast bacilli (AFB smear) has poor sensitivity (~10–20%) in routine practice ^7^. Culture has improved sensitivity (~50–60%) but is not widely available in many resource constrained settings and commonly takes at least 2–3 weeks for liquid culture growth, which is too slow to guide decision-making at the time of presentation ^8^.

In 2013, the World Health Organization endorsed the Xpert MTB/RIF (Xpert) assay (Cepheid, Sunnyvale, California, USA), a cartridge-based, polymerase chain reaction assay with a run time of 113 minutes, as the preferred initial test to investigate TB meningitis on the basis of a meta-analysis of 13 studies ^9^. Of the two major studies included in the meta-analysis, Patel and colleagues reported 67% sensitivity against microbiologically proven TBM and 36% against consensus clinical case definitions, while Nhu and colleagues showed 59% sensitivity against the same case definitions ^10– 12^. Additionally, use of a larger volume of centrifuged CSF improves sensitivity of Xpert ^10, 13^. Yet, inadequate negative predictive value means that a negative Xpert result has limited influence on clinical decision making ^14^.

There is evidence that use of Xpert for diagnosis of pulmonary TB reduces diagnostic delay, increases the rate of same day treatment, and decreases usage of empiric treatment ^15, 16^. However, for pulmonary TB, Xpert has not been shown to decrease mortality ^16– 18^. Yet, lessening diagnostic delay in persons with TBM may be more likely to lead to improved outcomes as compared to pulmonary TB given the high early mortality of TBM ^19^. Whether routine use of Xpert for investigation of suspected TBM has made an impact on diagnosis or mortality has not yet been investigated.

Herein we describe TBM diagnosis and outcomes over a 6.5-year period in prospective cohorts at two Ugandan referral hospitals.

## Methods

### Study population

Uganda is a high burden HIV setting, with a prevalence is 6.2% among adults aged 15 to 64 years with an estimated 60% viral load suppression in 2017 among all HIV-infected adults ^20^. Adults presenting with suspected meningitis (headache and neck stiffness +/- vomiting, fever, seizures, focal neurological deficits, or altered consciousness), to Mulago National Referral Hospital, Kampala, and Mbarara Regional Referral Hospital, were assessed for eligibility for enrolment in two consecutive randomised clinical trials investigating cryptococcal meningitis. The first trial Cryptococcal Optimal Antiretroviral Timing (COAT) investigated early versus delayed antiretroviral therapy in HIV-related cryptococcal meningitis ( www.clinicaltrials.gov: NCT01075152) and the second, Adjunctive Sertraline for the Treatment of Cryptococcal Meningitis (ASTRO-CM) evaluated whether sertraline when added to standard amphotericin-based therapy for cryptococcal meningitis, lead to improved survival ( NCT01802385). Screening began on 22 ^nd^ November 2010 and continued until 28 ^th^ May 2017. After an informed consent process for trial screening a diagnostic lumbar puncture was performed and baseline demographics and clinical information were recorded on all. Participants with non-cryptococcal meningitis were not enrolled into the clinical trials but followed until hospital discharge.

Any patient who received testing for TBM (CSF AFB smear, Xpert or mycobacterial culture) during this period was eligible to be included in the diagnostic accuracy analysis. Any patient who was ultimately treated for TBM was eligible to be included in one of the three TBM cohorts, from which data as used to compare rates of microbiological confirmation and outcomes. Cohort was determined by what type of TB testing they individual had undergone.

Microbiologically proven (definite) TB meningitis was defined as any positive AFB smear, culture or Xpert result from CSF testing. TBM treatment given in the absence of a positive microbiologic result, due to high index of clinical suspicion, was defined as ‘empiric TBM treatment’. Consensus uniform case definitions were used to categorise patients as definite, probable, possible or not TBM ^11^. TBM treatment included 12 months of antituberculous therapy with 6–8 weeks of adjunctive corticosteroids as per Ugandan guidelines ^21^.

### Cohort definitions and diagnostic tests used

**Figure 1.**
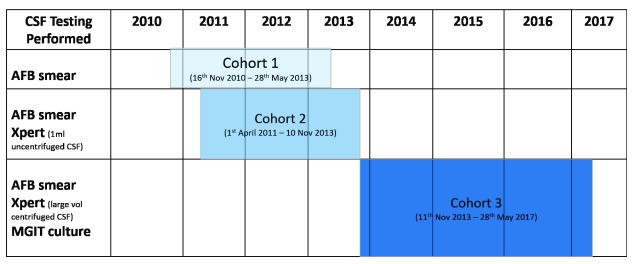
Timeline illustrating evolution of diagnostic testing.

Cohort one (16th November 2010 until 28 ^th^ May 2013) received only CSF AFB smear testing ( Figure 1). If available, 1mL cryopreserved CSF was later tested with Xpert MTB/Rif when Xpert became available. Cohort two (1 ^st^ April 2011 until 10 ^th^ November 2013) underwent CSF AFB smear and Xpert MTB/Rif on a 1ml sample of uncentrifuged CSF. Testing was performed at physician discretion when there was lymphocytic pleocytosis and/or high degree of clinical suspicion. In the period of overlap of cohort one and two (April 2011–May 2013), Xpert testing was not being done on a routine basis; subjects were included in cohort two when Xpert was done in real-time and in cohort one if Xpert was not done, or only done at a later date on cryopreserved specimens.

In cohort three (11 ^th^ November 2013 until 28 ^th^ May 2017) all cryptococcal antigen negative (IMMY, Norman, Oklahoma, USA) patients were systematically investigated for the presence of TB meningitis, irrespective of physician discretion. Subjects had comprehensive testing for TBM with CSF AFB smear (Mulago Hospital only), Xpert MTB/Rif on large volume centrifuged CSF ^13^ and CSF Mycobacteria Growth Inhibitor Tube culture (MGIT, Becton Dickinson, Franklin Lakes, USA). AFB smear was discontinued in Mbarara in 2013 as the sensitivity was deemed too low to justify further use. In patients with a confirmed diagnosis of cryptococcal meningitis (CM), if TBM co-infection was suspected, patients would be investigated for TBM at the physician’s discretion. 

### Assessment of outcome

In-hospital outcome was determined from case report forms, hospital medical records or follow-up telephone calls with the patient or their surrogate where hospital outcome was unknown. The outcome was categorised as discharged alive, deceased prior to hospital discharge or unknown (i.e. self-discharged against medical advice in an imminently terminal patient, hospital outcome undetermined, transferred to another facility).

### Statistical methods

Comparisons of categorical and continuous demographic and clinical characteristics by cohort were performed using Fisher’s exact tests and Kruskal-Wallis tests, respectively. Sensitivity of Xpert MTB/Rif was evaluated against a *composite reference standard* (any positive CSF test - AFB smear, Xpert or culture i.e. definite TBM according to the uniform case definition) ^11^. A separate analysis was conducted against the *uniform case definition* of probable or definite TBM ^11^. Concordance between Xpert MTB/Rif and culture was evaluated with a kappa statistic and McNemar’s test. Invalid tests (e.g. culture contamination, Xpert error) were counted as negative results. Mortality was first compared by cohort for participants with a known outcome using Fisher’s exact test. Data for patients with unknown outcome was imputed to assume first that 50% within each cohort died, or that 75% died (both within the expected mortality range for this population). Odds ratios and 95% confidence intervals were computed from multivariable logistic regression models with these imputed data, adjusted for 1) ART status, and 2) ART status and definite TBM diagnosis. Imputations were repeated with new random assignments to confirm results. Analyses were conducted using SAS version 9.4 (The SAS Institute, Cary, NC) and p-values <0.05 were considered statistically significant.

### Ethics

Institutional review board approvals for the studies and the associated screening process were obtained locally in Uganda [ASTRO: Mulago Hospital Research Ethics Committee (approval number, MREC 429); COAT: Makerere University School of Medicine Research and Ethics Committee (approval number, REC Ref No. 2009–022)], from the University of Minnesota (USA), and by the Uganda National Council of Science and Technology. Written informed consent for screening or participation in the studies was obtained from all participants or from their surrogates (e.g. family member or guardian) where the patient had altered mental status and did not have the capacity to provide consent.

## Results

### Participant characteristics

**Figure 2.**
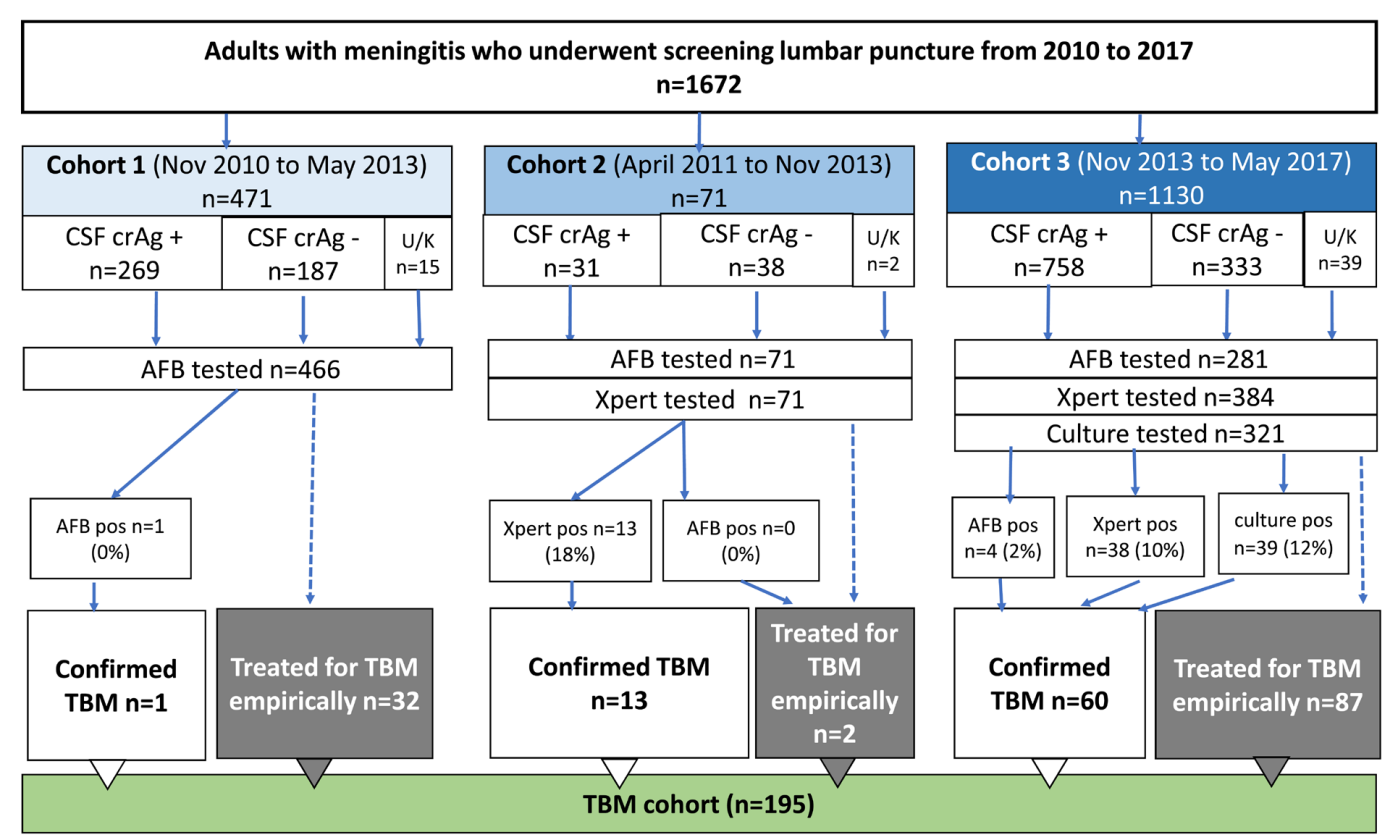
Illustration of flow of patients from the screening population into the TBM cohort.

Over the study period, 1672 patients with meningitis symptoms were assessed and underwent lumbar puncture: 1058 (63%) had a positive CSF cryptococcal antigen test, 558 (33%) had negative CSF cryptococcal antigen test (data missing, n=56). A total of 195 subjects were treated for TBM, see Figure 2. Overall 61% were male, median age was 35 years (IQR 30–42), 96% were HIV-positive, median CD4 count was 78 cells/μL (IQR 26–191) and the majority (69%) presented with British Medical Research Council severity grade II disease, see Table 1. Baseline characteristics were similar between cohorts with the exception of antiretroviral (ART) experience; 0% of participants were on ART in cohort one compared to 61% in cohort three (P<0.01).

Among the 74 cases of microbiologically proven TBM in this population with advanced HIV infection, 34% (25/74) had an acellular CSF (white cells <5 cells/μL) at presentation, and 4% (3/74) had a normal CSF profile (CSF cells <5 cells/μL, protein <45 mg/dL, and glucose >2.2mmol/l).

**Table 1.** Demographics, HIV details and outcomes of cohort.

	N with data	Cohort 1 Nov 2010 to May 2013	Cohort 2 Apr 2011 to Nov 2013	Cohort 3 Nov 2013 to May 2017		
Diagnostics used		AFB smear	AFB smear Xpert	AFB smear Xpert Culture	Total	P-value *
**N in TBM case cohort**		33	15	147	195	
Demographics						
**Sex**	195					0.58
Male		18 (55%)	8 (53%)	92 (63%)	118 (61%)	
**Age**	195					0.33
Median (IQR)		33 (29, 38)	35 (29, 40)	35 (30, 43)	35 (30, 43)	
HIV details						
**HIV status, n (%)**	195					1.00
HIV-positive		32 (97%)	15 (100%)	141 (96%)	188 (96%)	
**ART status, n (%)**	179					<0.01
On ART		0 (0%)	4 (27%)	80 (61%)	84 (47%)	
ART naive		32 (100%)	11 (73%)	52 (39%)	95 (53%)	
**CD4**	131					0.30
Median (IQR)		12 (7, 121)	148 (54, 169)	78 (26, 206)	78 (26, 191)	
TBM details						
**MRC severity grade,** **n (%)**	191					0.13
I		9 (27%)	4 (31%)	20 (14%)	33 (17%)	
II		22 (67%)	7 (54%)	102 (70%)	131 (69%)	
III		2 (6%)	2 (15%)	23 (16%)	27 (14%)	

*P-values from Fisher’s exact tests for categorical variables and Kruskal-Wallis tests for continuous variables.

### Method of diagnosis

Microbiological confirmation of TBM was made in 38% (74/195) of cases. The proportion of cases with microbiologically confirmed TBM (definite TBM) increased significantly, from 3% (1/33) in cohort one to 87% (13/15) in cohort 2 and 41% (60/147) in cohort 3 (P<0.01). Categorisation by uniform case definition is summarised in Table 2.

**Table 2.** Methods of Diagnosis.

	Cohort 1	Cohort 2	Cohort 3		
	AFB smear	AFB smear Xpert	AFB smear Xpert Culture	Total	P-value ^$^
All meningitis patients screened					
**Total number**	471	71	1130	1672	
Cryptococcal Antigen positive	269	31	758	1058	
Cryptococcal Antigen negative	187	38	333	558	
TBM diagnostic tests performed					
CSF AFB smear microscopy *					
N AFB performed	466	71	281	818	
N AFB positive	1 (0%)	0 (0%)	4 (2%)	5 (1%)	
CSF TB culture					
N TB culture performed	0	0	321	321	
N TB culture positive	0	0	39 (12%)	39 (12%)	
CSF Xpert MTB/Rif					
N Xpert performed (realtime)	0	71	384	455	
N Xpert positive	0	13 (18%)	38 (10%)	51 (11%)	
Uniform case definition					
Definite	1 (3%)	13 (87%)	60 (41%)	74 (38%)	<.01
Probable	5 (15%)	2 (13%)	11 (7%)	18 (9%)	
Possible	22 (67%)	0 (0%)	53 (36%)	75 (38%)	
Not	5 (15%)	0 (0%)	23 (16%)	28 (14%)	

Prior to November, 2013 any patient not prospectively tested with Xpert was considered in Cohort 1 *AFB smear was initially performed on all meningitis patients regardless of CSF Cryptococcal antigen result. From October 2013, it was only performed on those with a negative Cryptococcal antigen, and was later stopped altogether in Mbarara. ^$^P-value from Fisher’s exact test

There was a marked difference in physician threshold for empiric TBM therapy between the two clinical sites. In cohort three, Mulago Hospital recorded 44 cases of which 77% (34/44) were microbiologically confirmed and 23% (10/44) were empirically treated, whilst Mbarara Hospital recorded 103 cases of which 25% (26/103) were microbiologically confirmed and 75% (77/103) were empirically treated.

### Diagnostic accuracy of Xpert MTB/Rif

Xpert MTB/Rif was positive in 51 of 455 tested (11%), MGIT culture positive in 39 of 321 (12%) tested, AFB stain positive on 5 of 818 tested (1%), as summarised in Table 2.

Diagnostic accuracy of Xpert and MGIT were analysed in cohort three, when both assays were done routinely, and 60 participants had a microbiologically confirmed diagnosis (composite reference standard). Sensitivity of Xpert was 63% (38/60) against the *composite reference standard* and 54% (38/71) against the *uniform case definition* (probable or definite TBM). Sensitivity of MGIT culture was 65% (39/60) against the *composite reference standard* of definite microbiologic-confirmed TBM and 55% (39/71) against *uniform case definition* for probable or definite TBM.

**Figure 3.**
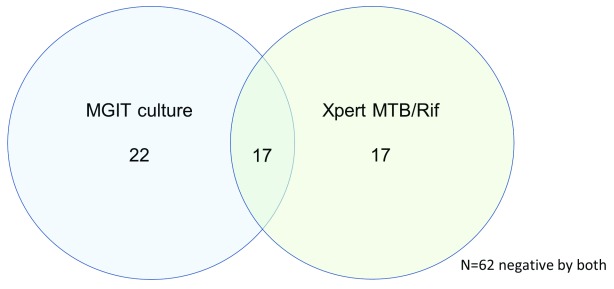
Venn diagram Illustrating the overlap of positive MGIT culture and Xpert test results in the n=118 samples tested with both assays. A total of 118 adults were tested with both MGIT culture and Xpert, of which 22 were positive by MGIT culture, 17 by Xpert and 17 by both tests. Neither test performed better than the other, p=0.423 by McNemar’s. A kappa statistics value of 0.23 95%CI [0.04, 0.41], p=0.01, suggests only slight agreement of the two assays.

Concordance between Xpert MTB/Rif and MGIT culture was analysed in the 118 with both Xpert and MGIT culture results available ( Table 3). Either Xpert or MGIT culture was positive in 56 patients, of which only 30% (17/56) were positive by both modalities (kappa 0.23 95% CI [0.04, 0.41], p=0.01) ( Figure 3). Neither method diagnosed significantly more cases than the other (p=0.42). 

**Table 3.** Summary of concordance between Xpert MTB/Rif and MGIT culture results.

Diagnostic Test		Xpert MTB/Rif		Total	P-value
		positive	negative		0.423
**MGIT culture**	positive	17	22	39	
	negative	17	62	79	
**Total**		34	84	118	

P-value from McNemar’s test N=118 (with both Xpert and culture results)

### Outcomes

**Figure 4.**
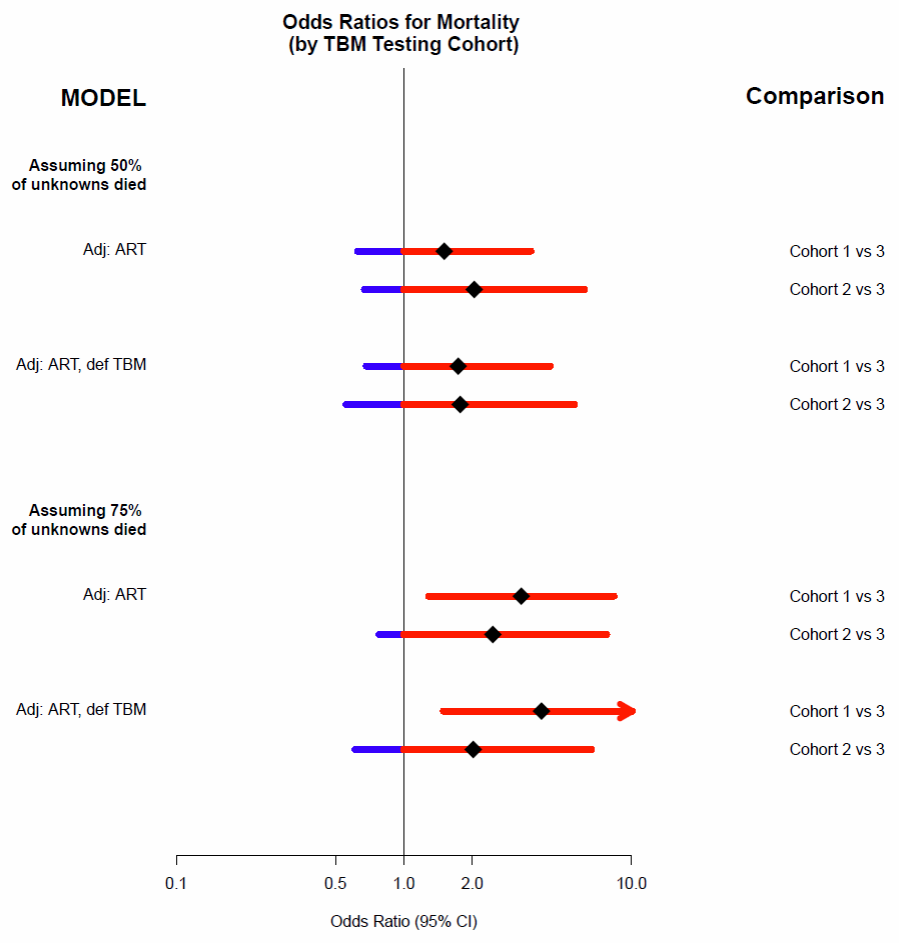
Illustration of odds of dying in cohort one and two compared to cohort three in a multivariate model. Odds ratios (and 95% confidence intervals) for death by the end of hospitalization comparing cohorts 1 and 2 to cohort 3, computed from multivariable logistic regression models with imputed data, adjusted for (1) ART status, and (2) ART status and definite TBM diagnosis. Data for patients with unknown outcome was imputed to assume that 50% within each cohort died, or that 75% died. In all models, neither ART nor definite TBM status had a significant association with in-hospital mortality, adjusted for cohort.

Hospital outcome was known for 142 participants, 53 had unknown outcomes or self-discharged against medical advice. Median time to death was 3 days (IQR 1–9 days) among those known to have died, and median length of hospitalization was 7 days (IQR 4–10 days) for participants known to have survived to hospital discharge. Among those with known outcomes, there was a non-significant decline in mortality from 57% in cohort one to 41% in cohort three (p=0.27) ( Table 4). Assuming that 50% of those with unknown outcome died, and adjusting for ART status and definite TBM diagnosis at hospitalization, the odds of dying were approximately twice as high for cohort one (aOR 1.7 95% CI [0.7, 4.4]) and cohort two (1.8 [0.6, 5.6]) as compared to cohort three. Assuming that 75% of those with unknown outcome died, adjusted odds of death increase further, cohort one (4.0 [1.5, 10.9]) and cohort two (2.0 [0.6,6.7]) compared to cohort three ( Table 4, Figure 4).

**Table 4.** Hospital outcomes.

	Cohort 1 Nov 2010 to May 2013	Cohort 2 Apr 2011 to Nov 2013	Cohort 3 Nov 2013 to May 2017		
Diagnostics used	AFB smear	AFB smear Xpert	AFB smear Xpert Culture	Total	P-value *
**N in TBM case cohort**	33	15	147	195	
Outcome of hospitalization					
Unknown	26 (79%)	4 (27%)	23 (16%)	53 (27%)	
Known	7 (21%)	11 (73%)	124 (84%)	142 (73%)	
Discharged Alive	3 (43%)	4 (36%)	73 (59%)	80 (56%)	0.27
Died	4 (57%)	7 (64%)	51 (41%)	62 (44%)	
Odds Ratio (Mortality) and 95% CI (on imputed data)					
** Assuming 50% of unknowns died**					
Adjusted for ART status	1.5 (0.6,3.6)	2.0 (0.7,6.2)	1		
Adjusted for ART status and confirmed TBM	1.7 (0.7,4.4)	1.8 (0.6,5.6)	1		
** Assuming 75% of unknowns died**					
Adjusted for ART status	3.3 (1.3,8.4)	2.5 (0.8,7.8)	1		
Adjusted for ART status and confirmed TBM	4.0 (1.5,10.9)	2.0 (0.6,6.7)	1		

Overall median (IQR) time in hospital was 7 (4, 10) days among those who were known to be discharged alive, and 3 (1, 9) days among those who were known to have died in hospital *P-value from Fisher’s exact test comparing KNOWN discharged alive vs KNOWN died; Odds ratios are the odds of being discharged alive, assuming 50% and 75% of those with unknown outcome died

## Discussion

Rapid molecular diagnostics have been predicted to reduce TB-related mortality ^22^ but little is reported about the impact of Xpert on TBM-related mortality. Here we report diagnosis and clinical outcomes among hospitalized Ugandans treated for TB meningitis over a 6.5-year period. In-hospital mortality was high in the cohort overall (44% 95% CI [36,52%]), similar to other research settings with high HIV prevalence ^7, 19, 23, 24^. The adjusted model found that odds of in-hospital mortality were almost two-fold higher in the earliest cohort, tested by CSF smear microscopy only, compared to that of the most recent cohort in which Xpert (and culture) were routinely performed. Though severity of TBM at presentation was similar over the study period and TBM treatment recommendations have not changed for Uganda, there are multiple other potential confounding factors due to the nature of the study. Improved access to ART, strengthening in healthcare services, increased awareness of TBM amongst communities and healthcare workers or changes in employment of empiric treatment all may have occurred over the study period and if present, may have impacted our findings. Due to these potential confounding factors, it is not possible to draw a conclusion about whether Xpert has reduced TBM mortality in this setting.

The proportion of ART experienced subjects increased significantly over time with improved access to ART treatment in Uganda and because the parent trial in cohort one enrolled only ART naïve subjects ^25^. Although ART status was not associated with mortality, we adjusted for ART in multivariable models due to the large discrepancy in ART status between cohorts. Nonetheless the impact of ART use is likely to have played a role in the observed decline in mortality.

Despite a non-significant decline in mortality, a current case-fatality rate of 41% remains unacceptably high and highlights the remaining work required to achieve the WHO goal of reducing TB-related deaths by 90% by 2030 ^26^. Initiating treatment in the early stage of disease is the single most important factor in improving outcomes ^7^. Earlier presentation to the hospital is essential for prompt diagnosis and treatment initiation, yet, 83% of our cohort presented with MRC grade II or III disease.

Once the patient presents to care, an affordable, rapid, and reliable test that can effectively confirm or rule out TBM is crucial for prompt diagnosis. In this predominantly HIV-positive TBM cohort, sensitivity of Xpert was 63% against the composite reference standard. Thus, even though results were available rapidly, Xpert missed over one in three cases. The next generation assay Xpert MTB/Rif Ultra has an analytic limit of detection of 15 colony forming units (CFU)/ml, compared to 113 CFU/ml for Xpert ^27^. Ultra appears to be significantly more sensitive than Xpert or culture for the diagnosis of TBM (95% versus 45% and 45% respectively, P<0.001) ^28^. Whether Ultra can reduce diagnostic delay and improve outcome from TBM requires further prospective evaluation.

Where both Xpert and MGIT had been done, less than a third (23%, 17/74) of confirmed cases were positive by both modalities. This is consistent with prior findings and is likely due to the relatively higher sensitivity of culture versus Xpert, and the ability of Xpert to detect non-viable TB bacilli damaged by host-immune response and/or antituberculous drug therapy ^13, 28^. Neither test performed better than the other (P=0.42) and currently CSF culture continues to earn a place in TBM diagnostics testing algorithms.

Until a rapid test with suitable negative predictive value is widely available, there is likely to be on-going heterogeneity in clinical practice regarding frequency of empiric TBM therapy (TB treatment in the absence of a positive result). Here, Mulago Hospital participants were treated for TBM on an empiric basis in under one quarter of cases as opposed to over three quarters of cases at Mbarara Hospital, despite the same diagnostic armamentarium being available in both settings. In our study settings, it is likely that individual or departmental clinical practice and risk thresholds for TB treatment initiation dictate this variation. Though empiric TBM therapy is potentially life-saving, significant risks such as side effects, drug-interactions and adjunctive steroids in an already immunosuppressed population need to be considered. Ideally, a rapid, accurate test allows therapy for TB meningitis to be started promptly only in those who actually have TBM. Overall, the proportion with microbiologically confirmed TBM increased significantly from 3% in cohort one to 41% in cohort three (P<0.01). In cohort two, selection bias could have impacted on results as Xpert was only performed in cases where there was extremely high index of suspicion and empiric treatment was given only twice in those with a negative Xpert (4%, 2/56). The low number of empiric diagnoses during this period were likely due to over-confidence in Xpert’s ability to rule-out TBM. As understanding regarding the limitations of Xpert for the diagnosis of TBM became known, empiric TBM treatment rose ^14^.

Limitations of this study relate to the nature of available data namely missing data on hospital and long-term outcomes, the time to starting TB treatment, unbalanced numbers in each cohort, and other un-adjusted confounding factors and potential sources of bias. When imputing data in the model we assumed that either 50% or 75% of patients with unknown outcome actually died, which is a clinically reasonable judgment for this population ^4^.

Here we present important data on diagnostic confirmation and TBM mortality during a period of TB diagnostic evolution. There has been a significant increase in microbiological confirmation and a modest, albeit non-significant, decline in mortality since introduction of Xpert and culture in our study setting. An on-going multifaceted approach is needed to further reduce death and disability from TBM.

## Consent

Written informed consent for publication of the anonymised data was obtained from the participants or their surrogates.

## Data availability

The database contains individual level data and as such is not available through an open-access data repository. The database is stored on a secure server at University of Minnesota. Researchers interested in accessing the data can contact the corresponding author (FVC), the last author (DRB) or the Division of Biostatistics at the University of Minnesota. Data access will be granted to active researchers in the field with the agreement of the authors.
