# Supplementary material for: Tuberculous meningitis diagnosis and outcomes during the Xpert MTB/Rif era: a 6.5-year cohort study in Uganda
Source: Wellcome Open Res. 2018 Jul 3;3:64. Originally published 2018 May 29. [Version 2] doi: 10.12688/wellcomeopenres.14610.2 (PMC6039934; doi:10.12688/wellcomeopenres.14610.2)
Supplement: Peer review history

# Peer review: Referee response for version 2

**DOI:** 10.21956/wellcomeopenres.15994.r33439

**Published:** 2018-07-31

**Authors:** Raph L. Hamers

**Affiliations:** 1 Eijkman-Oxford Clinical Research Unit, Jakarta, Indonesia

The authors have done a great job incorporating the reviewers' comments, which has generally improved the manuscript. I have no further comments or concerns.

I have read this submission. I believe that I have an appropriate level of expertise to confirm that it is of an acceptable scientific standard.

# Peer review: Referee response for version 2

**DOI:** 10.21956/wellcomeopenres.15994.r33441

**Published:** 2018-07-12

**Authors:** Angharad Davis, Sean Wasserman

**Affiliations:** 1 National Hospital for Neurology and Neurosurgery, University College London Hospitals, London, UK; 2 University of Cape Town, Cape Town, South Africa

I have read through the comments. Many thanks to the authors. We are happy that the points have been sufficiently addressed. We are happy that the paper should be accepted and no more changes are required.

We have read this submission. We believe that we have an appropriate level of expertise to confirm that it is of an acceptable scientific standard.

# Peer review: Referee response for version 2

**DOI:** 10.21956/wellcomeopenres.15994.r33440

**Published:** 2018-07-05

**Authors:** Tom Boyles

**Affiliations:** 1 Wits Reproductive Health and HIV Institute, University of the Witwatersrand, Johannesburg, South Africa

I am satisfied that the authors have adequately addressed the points I made in my review.

I have read this submission. I believe that I have an appropriate level of expertise to confirm that it is of an acceptable scientific standard.

# Peer review: Referee response for version 1

**DOI:** 10.21956/wellcomeopenres.15907.r33229

**Published:** 2018-06-15

**Authors:** Angharad Davis, Sean Wasserman

**Affiliations:** 1 University of Cape Town, Cape Town, South Africa; 2 National Hospital for Neurology and Neurosurgery, University College London Hospitals, London, UK

Many thanks for requesting us to review this article which addresses an important unanswered question in TB diagnostics. We have made specific points regarding each section of the manuscript as well as giving an overall impression. 

 This prospective cohort studies observes differences in diagnosis and hospital outcomes related to TBM across three cohorts of patients recruited over a 6.5 year period in Uganda. The study aims to measure the impact on diagnosis and hospital outcomes with the addition of Gene Xpert testing or Gene Xpert *and*routine liquid media culture to standard diagnostic testing (CSF smears) over time. 

Abstract

- The study design is unclear: is this a retrospective or post-hoc analysis of data collected from other studies? The nature of the parent studies should be mentioned.
- The denominator is not clearly reported: the inclusion criteria for the current analysis appears to be “all meningitis suspects with a negative CSF cryptococcal antigen.” Were 558 patients with negative CSF cryptococcal antigen test included in the analysis?
- The conclusion that “in-hospital mortality has declined” seems exaggerated because this trend towards lower mortality over the study periods was not significant, and may have been due to other factors unrelated to changing diagnostics.

 

Methods

- The inclusion criteria should be clearly defined and justified.
- Differences between the cohorts could have introduced bias towards improved outcomes (type 1 error): cohorts 1 and 2 at physician discretion, cohort 3 were systematically investigated. This should be addressed in the discussion.
- Please explain how the performance of a test (eg Xpert) can be compared to a reference standard that includes the test itself (“any positive CSF test - AFB smear, Xpert or culture”)?
- Were the variables in the multivariate model selected *a priori*?
- Was ethics permission obtained for this sub-study/analysis?

 

Results

- The denominator is not clearly reported: the inclusion criteria for the current analysis appears to be “all meningitis suspects with a negative CSF cryptococcal antigen.” Were 558 patients with negative CSF cryptococcal antigen test included in the analysis, or was it 195 subjects that were treated for TBM? This should match the inclusion criteria, which appear to be any patient with suspected meningitis and negative CrAg testing.
- A large proportion of patients in cohorts 2 and 3 were on ART at the time of presentation: this could alter their prognosis and bias the results. Although the model adjusted for this, it should be emphasised more in the discussion.
- There is a discrepancy in the number of microbiologically proven cases: 
  - “Among the **76**cases of microbiologically proven TBM”
  - “Microbiological confirmation of TBM was made in 38% ( **74**/195) of cases”
- Why was the number of confirmed cases so high in Cohort 2?
- The paragraph describing empiric treatment practices requires clarification: how was ‘empiric treatment’ defined? The numbers appear to suggest that no patients with a confirmed diagnosis were empirically treated (ie empiric treatment = no microbiological confirmation). Suggest changing the terminology to make this clearer – 1. Treated based on positive result; 2. Treated without a positive result. It seems that in Mulago Hospital a higher proportion of treated patients had positive results, and therefore did not require ‘empiric treatment.’ So, the important question seems to be why there was such a bug discrepancy in the number of confirmed cases between the two sites, allowing ‘empiric treatment’ to be avoided?
- The denominator is again unclear for the diagnostic accuracy section: where do the “455 tested” come from if only 195 cases were included in the study (or is it 558?). Suggest including a consort diagram to explain the patient populations for this study.
- Suggest including a 2 x 2 table to demonstrate the performance of diagnostic tests. Although the sensitivity of Xpert is 63% compared to a reference standard, this this standard is only present in 40% of cases (60/147) in Cohort 3. 

 

Discussion

- See comments related to discussion above
- The main (and probably only data-driven) conclusion is that more confirmed TBM cases were detected with the use of Xpert and culture. The authors did not convincingly show an improvement in outcomes. Any observed trends could be secular, or related to other confounding factors resulting from different populations, selection criteria, management practices, etc. 

 

 The study observes three cohorts recruited from two parent trials. Although the data provides an important insight into the unanswered questions above, the suitability of the cohorts as comparators to one another does pose some limitation to how the results can be interpreted. Namely there is large variation between the observed cohorts in a number of aspects namely the size of cohorts, ART status, and availability of follow up data. Although ART status had been accounted for in analysis, the impact of absent follow up data which is much more prominent for cohort 1 (79%) compared to cohort 3 (16%) should be made clearer in the discussion. Also, how were the patients allocated to cohort 1 and 2 during the overlapping study period (1 ^st^April 2011 until 28 ^th^May 2013)? Were those where there was more diagnostic doubt allocated to receive testing with GeneXpert (and therefore subsequently included in cohort 2 than cohort 1?).. could this have affected outcomes observed between the two groups?

 It is possible that apart from the mentioned variables, that over time, other aspects may have influenced hospital outcome in these patients over a period where there has naturally been some advances made in TBM care besides the diagnostics discussed. For example the use of imaging diagnostics, time to treatment, availability of drugs, better understanding of supportive measures in TBM. I feel that these should form part of the discussion here and although they may not be quantifiable in this setting, should be acknowledged as possible factors for the observed differences over time. In this setting specifically were there changes in resource allocation? Did hospital facilities change?

We have read this submission. We believe that we have an appropriate level of expertise to confirm that it is of an acceptable scientific standard, however we have significant reservations, as outlined above.

# Response

**Published:** 2018-06-26

**Authors:** Fiona Cresswell

**Affiliations:** London School of Hygiene & Tropical Medicine, UK

Thank you Dr Wasserman and Dr Davis for your many comments, questions and suggestions, which we will address this individually: 

**Abstract**

**1. The study design is unclear: is this a retrospective or post-hoc analysis of data collected from other studies? The nature of the parent studies should be mentioned.**

The nature of the parent studies has been expanded in the methods section of the revised manuscript. We have also added further information to the abstract and clarified that this is a post-hoc analysis of prospective cohorts. 

**2. The denominator is not clearly reported: the inclusion criteria for the current analysis appears to be “all meningitis suspects with a negative CSF cryptococcal antigen.” Were 558 patients with negative CSF cryptococcal antigen test included in the analysis?**

The main analysis for microbiological confirmation and mortality relates to the 195 people within the three TBM prospective cohorts. Of the 1672 screened any patient who received any testing for TBM (CSF AFB smear, Xpert or mycobacterial culture) during this period was eligible to be included in table 2 and the diagnostic accuracy of Xpert and culture analysis. Any patient who was ultimately treated for TBM (n=195) was eligible to be included in the three prospective TBM cohorts, and it was this group on whom the rate of microbiological confirmation and outcomes were compared. We have added a schematic diagram to the manuscript which i hope clarifies the nature of the study population.  

**3. The conclusion that “in-hospital mortality has declined” seems exaggerated because this trend towards lower mortality over the study periods was not significant, and may have been due to other factors unrelated to changing diagnostics**

You are absolutely correct. We have changed to text in the abstract to read "Since 2010, as TBM diagnostics have evolved, microbiologically-confirmed TBM diagnoses have increased and there has been a non-significant decline in TBM in-hospital mortality. Due to multiple possible confounding factors it is not possible to conclude what has driven this decline in mortality". 

**Methods**

**1. The inclusion criteria should be clearly defined and justified**

The following text has been added to the revised manuscript:

"Adults presenting with suspected meningitis (headache and neck stiffness +/- vomiting, fever, seizures, focal neurological deficits, or altered consciousness), to Mulago National Referral Hospital, Kampala, and Mbarara Regional Referral Hospital, were assessed"  

"Participants with non-cryptococcal meningitis were not enrolled into the clinical trials but followed until hospital discharge". 

"Any patient who was ultimately treated for TBM was eligible to be included in one of the three TBM cohorts, on which rates of microbiological confirmation and outcomes were compared. Cohort was determined by what type of TB testing they had undergone". 

**2. Differences between the cohorts could have introduced bias**

This is a valid observation. Text stating "selection bias could have impacted on results" has been added to the discussion. 

**3. Please explain how the performance of a test (eg Xpert) can be compared to a reference standard that includes the test itself (“any positive CSF test - AFB smear, Xpert or culture”)?**

Whilst we acknowledge this is an imperfect reference standard there is not a suitable reference standard that can be used in TBM diagnostic accuracy studies. Importantly, each of the tests is know to have a high specificity so the chance of false positives is extremely low, especially in a symptomatic population with a high disease prevalence. Latent class analysis would be a potential statistical approach that could be used in such cases which lack a perfect reference standard, and we would be interested to do such an analysis in future. 

**4. Were the variables in the multivariate model selected *a priori*?**

Yes

**5. Was ethics permission obtained for this sub-study/analysis?**

Yes, the informed screening consent process sought approval for both storage of specimens for future research and use of data for analyses / research relating to meningitis. 

**Results**

**1. The denominator is not clearly reported: the inclusion criteria for the current analysis appears to be “all meningitis suspects with a negative CSF cryptococcal antigen.” Were 558 patients with negative CSF cryptococcal antigen test included in the analysis, or was it 195 subjects that were treated for TBM? This should match the inclusion criteria, which appear to be any patient with suspected meningitis and negative CrAg testing.**

I hope the response to comment on abstract has clarified this. 

**2. A large proportion of patients in cohorts 2 and 3 were on ART at the time of presentation: this could alter their prognosis and bias the results. Although the model adjusted for this, it should be emphasised more in the discussion.**

This has been emphasised in the discussion of the revised manuscript. 

**3. There is a discrepancy in the number of microbiologically proven cases**

Thanks for spotting this typo. This has been corrected to 74. 

**4. Why was the number of confirmed cases so high in Cohort 2**

The review by Dr Hamers also made this comment. Please see response to his comment for a potential explanation. 

**5. The paragraph describing empiric treatment practices requires clarification: how was ‘empiric treatment’ defined? The numbers appear to suggest that no patients with a confirmed diagnosis were empirically treated (ie empiric treatment = no microbiological confirmation). Suggest changing the terminology to make this clearer.**

Thank you. We have defined this more clearly in the methods. 

**6. So, the important question seems to be why there was such a big discrepancy in the number of confirmed cases between the two sites, allowing ‘empiric treatment’ to be avoided?**

Both hospitals have the same available diagnostics so i believe the difference in empiric treatment relates to local or personal thresholds applied to the initiation of TB treatment in the absence of a confirmatory test. You will no doubt have observed different treatment thresholds amongst colleagues, especially faced with a critically ill patient. As Mbarara Hospital is a relatively small hospital with good longterm retention of staff, especially in the clinical research setting, it is possible that a handful of clinicians may have seen the majority of TBM cases and been more willing to initiate TB treatment despite negative tests than in Kampala where there is a bigger team and clinicians rotate regularly.   

**7. Suggest including a consort diagram to explain the patient populations for this study.** 

Thanks for this suggestion. We have included a schematic of patient flow from the screening population into the cohort. It is not a classic consort diagram since this population were not part of an RCT. 

**8. Suggest including a 2 x 2 table to demonstrate the performance of diagnostic tests.**

We have added a 2x2 table. 

**Discussion**

**1. The main (and probably only data-driven) conclusion is that more confirmed TBM cases were detected with the use of Xpert and culture. The authors did not convincingly show an improvement in outcomes. Any observed trends could be secular, or related to other confounding factors resulting from different populations, selection criteria, management practices, etc.**

Indeed. We acknowledge the limitations of the data available to us and have tempered the conclusion accordingly in the revised manuscript. 

**2. For example the use of imaging diagnostics, time to treatment, availability of drugs, better understanding of supportive measures in TBM. I feel that these should form part of the discussion here and although they may not be quantifiable in this setting, should be acknowledged as possible factors for the observed differences over time. In this setting specifically were there changes in resource allocation? Did hospital facilities change?**

This a good thoughts and we concede that many potential confounders exist. However, we could show from our limited data that severity at presentation across cohorts was the same. Whilst the health services in the private sector have evolved in this time period the government facilities remain under resourced without access to routine blood tests and imaging. Thankfully access to TB medication has been stable for many years. Healthcare worker practices may certainly have changed and we recognise this as a potential confounder. 

Thank you again for your detailed review. I hope the responses and amendments are satisfactory.

# Peer review: Referee response for version 1

**DOI:** 10.21956/wellcomeopenres.15907.r33231

**Published:** 2018-06-11

**Authors:** Raph L. Hamers

**Affiliations:** 1 Eijkman-Oxford Clinical Research Unit, Jakarta, Indonesia

This is a large prospective study that assesses TBM diagnostics and mortality in 2 reference hospitals in Uganda, including 3 HIV adult cohorts that can be distuinguished by the routine use of different diagnostic testing approaches on CSF: 1) AFB only (2010-2013); 2) AFB+Xpert (uncentriguged CSF) (2011-2013); 3) AFB+Xpert (centriguged, large volume)+MGIT (2013-2017). The study is a commendable effort to improve care and outcomes of TBM in LMIC. It is well-written and presents unique and rich data that are very informative to clinicians who seek to improve outcomes of TBM. The authors report substantial achievements in improving access to and performance of TBM diagnostics.

 Major comments

 1. The title suggests that the aim of the study was to investigate if better TBM diagnostics can lead to improved survival. After reading the article, this question cannot be answered. The authors rightfully formulate their conclusion more prudently: “As TBM diagnostics have improved, microbiologically-confirmed TBM diagnoses have increased and in-hospital mortality has declined”. Indeed, many other factors could have influenced the differential outcomes across the 3 historical cohorts that span the time period of 2011-2017, and I feel the authors could present these complex factors in a more structured and comprehensive manner in the Discussion:

 - For instance, the main driver of the difference observed is the huge discrepancy between ART status across cohorts (0-61% on-ART) -is it at all possible to adjust for this major difference with statistical methods alone? 

 - The authors note no major changes in treatment practices over time, although they also report substantial differences between the 2 hospitals in ability to confirm diagnosis and start empirical treatment, which may have impacted on outcomes (if varied with time).

 - Referral practices and general awareness among care providers may have improved in the study setting (the authors note they do not have data on time to Rx initiation), especially given that the authors have implemented 2 large clinical trials in the same period.

 - The patient case mix may have changed over time in many other ways (e.g. better nutrition, better education, lower TB incidence, better HIV diagnosis and treatment, etc)

 My recommendation is to reword the title, to avoid misleading the readership.

 2. The authors have (deliberately) done separate analyses on the 3 cohorts, and combining historical cohorts may have its limitations. Nonetheless, the combined cohort offers opportunities to attempt answering the main question. In this respect, it would be interesting if the authors could undertake a multivariate analysis of the combined cohort data to identify what are the main determinants of mortality (adjusting for time period). This could help to establish whether indeed the diagnostics influenced outcomes, or that the survival gain was mainly driven by other factors (e.g. ART). In my view, adding this analysis could strengthen the paper.

 Minor points

 1. The % of microbiological confirmation is 3% in cohort #1, 87% in cohort #2, and 41% in cohort #3. It would be relevant to learn why the latest % seems to be lower than the middle one (despite better diagnostic protocols).

 2. The degree of agreement between Xpert and MGIT is very low (kappa 0.23). I would like to see a better explanation for this discrepancy, and how this knowledge can to be applied in recommendations for testing algorithms in practice.

I have read this submission. I believe that I have an appropriate level of expertise to confirm that it is of an acceptable scientific standard, however I have significant reservations, as outlined above.

# Response

**Published:** 2018-06-26

**Authors:** Fiona Cresswell

**Affiliations:** London School of Hygiene & Tropical Medicine, UK

We thank Dr Hamers for his insightful and helpful comments. 

**1. The title suggests that the aim of the study was to investigate if better TBM diagnostics can lead to improved survival. After reading the article, this question cannot be answered.**

 In light of your observation we have changed the title to "Tuberculous meningitis diagnosis and outcomes during the Xpert MTB/Rif era: a 6.5-year retrospective cohort study in Uganda". We hope this better reflects what can actually be concluded from this retrospective data analysis. 

**2. Indeed, many other factors could have influenced the differential outcomes across the 3 historical cohorts that span the time period of 2011-2017, and I feel the authors could present these complex factors in a more structured and comprehensive manner in the Discussion.** 

 Thank you for this suggestion. We have taken on board and revised the discussion to acknowledge the multiple potential unadjusted confounding factors and the inability to draw a conclusion about the impact of diagnostics on outcomes from the data we have available. 

**3. The authors have (deliberately) done separate analyses on the 3 cohorts, and combining historical cohorts may have its limitations. Nonetheless, the combined cohort offers opportunities to attempt answering the main question. In this respect, it would be interesting if the authors could undertake a multivariate analysis of the combined cohort data to identify what are the main determinants of mortality (adjusting for time period). This could help to establish whether indeed the diagnostics influenced outcomes, or that the survival gain was mainly driven by other factors (e.g. ART). In my view, adding this analysis could strengthen the paper.**

 Thank you for this suggestion. Assessing risk factors for mortality was not the primary intent of the paper but we agree this would make an interesting analysis in future. The TBM testing cohorts in this study generally represent testing time period and cohort is the primary variable of interest for this study. Since ART use changed over time and definite TBM is associated with increased risk of mortality, these covariates were chosen to be included in adjusted analyses. Mortality was unknown for 27% of participants, so we conducted a series of models with imputations assuming that 50% within each cohort died, or that 75% died (both within the expected mortality range for this population).  Six multivariable models of in-hospital mortality were run and all cohorts were included in all models. Cohort 3 was chosen as the reference category for presenting the odds ratios of interest since it represents the most current testing era. In all models, neither ART nor definite TBM status had a significant association with in-hospital mortality. All models run include all cohorts and the odds ratios comparing cohorts from the adjusted models are presented in Figure 4 in the (revised) manuscript.

**4. The % of microbiological confirmation is 3% in cohort #1, 87% in cohort #2, and 41% in cohort #3. It would be relevant to learn why the latest % seems to be lower than the middle one (despite better diagnostic protocols).**

 This is an interesting observation and we believe relates to two factors:

 a) In cohort 2, during the initial period after Xpert introduction, it was only being used on cases with an extremely high clinical index of suspicion so there was a selection bias in population tested. 

 b) The % of confirmed case amongst the total number treated for TBM in cohort 2 appears spuriously high as the amount of empiric treatment was very low during the cohort. One potential reason is that there was an over confidence in Xpert's ability to be able to rule out TBM. In subsequent years a number of papers showed that the negative predictive value of Xpert is such that a negative test result should not deter TB treatment and empiric treatment was used more frequently again in out setting. 

**5. The degree of agreement between Xpert and MGIT is very low (kappa 0.23). I would like to see a better explanation for this discrepancy, and how this knowledge can to be applied in recommendations for testing algorithms in practice.**

 This is another interesting observation, and a somewhat surprising finding to us also. We have not elaborated in the discussion in the interest of brevity but possible explanations are mentioned here:

**Positive on Xpert whilst negative on culture:**

 We lack data about the number of cases in the cohort who were on TB treatment at the time of lumbar puncture. It is plausible that if TB treatment had been initiated in the days or weeks prior to the CSF analysis the mycobacterium may have been rendered non-viable for culture. In our setting many patients present with a disseminated TB picture, some of whom have been coughing for several weeks prior to development of neurological symptoms and already initiated on antituberculous therapy from outpatient settings. We hope to capture this data more comprehensively in prospective studies.  

**Positive on culture whilst negative on Xpert:**

 Culture has a lower limit of detection that Xpert so patients with bacillary burdens on the <100CFU/ml region would likely be identified by culture only. 

**Considerations for future testing algorithms:**

 It is hopeful that the next generation assay Xpert MTB/Rif 'Ultra' will have a limit of detection similar to that of culture, so given that it can also detect non-viable bacilli, it is likely to perform better the culture in the diagnosis of TBM (as was the case in Bahr N. Lancet ID. 2018). 

 Testing algorithms must be customised and take into consideration the setting, HIV-prevalence, cost and the volume of CSF available for testing, amongst other things. Splitting a small CSF sample between multiple assays is likely to be counterproductive. In our setting we would recommend Ultra as the initial CSF test for TBM (in patients with a negative CSF cryptococcal antigen lateral flow assay). Culture remains an important adjunctive test especially in patients where there is a risk of drug resistance. 

 We have submitted a revised manuscript and hope these changes and responses are to Dr Hamer's satisfaction.

# Peer review: Referee response for version 1

**DOI:** 10.21956/wellcomeopenres.15907.r33230

**Published:** 2018-06-06

**Authors:** Tom Boyles

**Affiliations:** 1 Wits Reproductive Health and HIV Institute, University of the Witwatersrand, Johannesburg, South Africa

Thank you for the opportunity to review this article which describes the investigation, treatment and outcomes of a cohort of mostly HIV infected adults in Uganda who were suspected of having TB meningitis. It is well written and easily reproducible by others.

 The title asks the question of whether improved diagnostics can reduce mortality in TBM. It might be better to say ‘Have improved diagnostics improved outcomes?’ My view of the data is that it provides very limited evidence that improved diagnostics have improved outcomes of TBM in this cohort. The small improvements in outcome over time are statistically non-significant and due to the observational design are likely to be influenced by numerous unmeasured confounding factors. For example, the experience of clinician may well have changed over time, we know that empiric treatment was used differently in the 2 centres but it might also have changed over time. Part of the work-up of a patient with suspected TBM is a search for extra-neural TB and we do not know how this changed over time although it is likely that Xpert MTB/RIF was used for non-CSF samples in later cohorts compared to earlier, the same might be said for urine LAM.

 My view as a reader is therefore that this data does not provide convincing evidence that the change in diagnostics was the driver of the small changes in mortality. It must be remembered that new diagnostics also have the potential to worsen outcomes, particularly if clinicians miss-interpret negative tests as ruling out the condition as might well occur with Xpert MTB/RIF and TBM.

 I think that this well written work definitely deserves to be published but feel that the conclusions should reflect a greater level of uncertainty.

 Minor points:

 The abstract says that all 1672 patients were HIV infected but in the results section is says 96% were HIV infected so there is a discrepancy.

 Last line, para 1 of introduction, probably remove word ‘to’.

 Last line. Para 3 of introduction- It is not that Xpert cannot substitute for clinical judgement- Xpert is used to enhance clinical judgement, it’s just that the  poor sensitivity means that when negative it has limited influence on decision making

 Statistical methods- the uniform case definition is probable or definite TBM- do the authors think this is a reasonable reference standard for other tests? My view is that the threshold for treating TBM is very low and that patients with possible/probably or definite TBM should receive treatment and therefore this would be a more appropriate reference standard- what are your views on that?

 Methods- Not clear if there were 76 or 74 microbiologically proven cases

 Discussion- Para 1, repeat of ‘that of’

 Discussion- Para 4, First sentence implies that tests are the only answer to the diagnosis of TBM, what about clinical prediction rules relying on clinical data- although so far they have not been very successful, neither have tests so a robust CPR may negate the need for tests.

 Discussion- Para5, Do the authors really think there are dead bacilli in CSF? Some of the authors have argued the opposite in their recent paper on Xpert Ultra so you can’t have it both ways.

I have read this submission. I believe that I have an appropriate level of expertise to confirm that it is of an acceptable scientific standard, however I have significant reservations, as outlined above.

# Response

**Published:** 2018-06-26

**Authors:** Fiona Cresswell

**Affiliations:** London School of Hygiene & Tropical Medicine, UK

Dear Dr Boyles, 

 Thank you for providing a considered and constructive of our paper. I'd like to respond to each comment in turn. 

**1. The findings provide limited evidence that improved diagnostics have improved outcomes of TBM in this cohort and due to the observational design are likely to be influenced by numerous unmeasured confounding factors. I think that this well written work definitely deserves to be published but feel that the conclusions should reflect a greater level of uncertainty.**

 We acknowledge the limitations of the retrospective data we present and the inability to account for a multitude of other confounding factors. The title of the manuscript has been changed to "Tuberculous meningitis diagnosis and outcomes during the Xpert MTB/Rif era: a 6.5-year retrospective cohort study in Uganda". In the conclusion of the revised manuscript we given more emphasis to uncontrolled confounding factors the fact that the data on impact of improved diagnostics on outcomes in inconclusive. 

**2. Part of the work-up of a patient with suspected TBM is a search for extra-neural TB and we do not know how this changed over time although it is likely that Xpert MTB/RIF was used for non-CSF samples in later cohorts compared to earlier, the same might be said for urine LAM.**

 Indeed, sputum samples may be undergone testing with Xpert to assist in the diagnosis of TBM in  cohort 3. TB-LAM has only become available in 2018 in Uganda so this will not have impacted our study which concluded in May 2017. 

**3. It must be remembered that new diagnostics also have the potential to worsen outcomes, particularly if clinicians miss-interpret negative tests as ruling out the condition as might well occur with Xpert MTB/RIF and TBM.**

 I completely agree. The delay in waiting for a diagnostic test result must also be recognised, which can be several days in many hospital settings. Bring on an accurate bedside POCT. 

**4. ​​​​​​​The abstract says that all 1672 patients were HIV infected but in the results section is says 96% were HIV infected so there is a discrepancy.**

 Thanks for pointing out this oversight. I have added the word predominantly to the abstract. 

**5. Last line, para 1 of introduction, probably remove word ‘to’.**

 Well spotted. Thank you. 

**6. Last line. Para 3 of introduction- It is not that Xpert cannot substitute for clinical judgement- Xpert is used to enhance clinical judgement, it’s just that the  poor sensitivity means that when negative it has limited influence on decision making.** 

 The text of the revised manuscript has been changes to read "a negative Xpert result has limited influence on clinical decision making". 

**7. ​​​​​​​Statistical methods- the uniform case definition is probable or definite TBM- do the authors think this is a reasonable reference standard for other tests? My view is that the threshold for treating TBM is very low and that patients with possible/probably or definite TBM should receive treatment and therefore this would be a more appropriate reference standard- what are your views on that?**

 This is a good discussion point. Where is the correct place to draw the line? The HIV co-infection makes this particularly challenging as CMV meningoencephalitis, toxoplasmosis, PML etc can muddy the water further. In Uganda, in our HIV/TBM cases 1/3rd have CSF WBC<5 which can mean the points scored in the CSF category are fewer. Furthermore in resource constrained settings access to neuroimaging and extra neural sampling can be limited which again affects the ability to fully characterise the case. It would be completely acceptable to include 'possible' in the reference standard but we chose 'probable and definite' as we were concerned that the 'possibles' may include other HIV-realted neuropathologies. 

**8. ​​​​​​​Methods- Not clear if there were 76 or 74 microbiologically proven cases**

 Thanks for pointing out this typo. The test has been corrected to 74. 

**9. Discussion- Para 1, repeat of ‘that of’**

 Well spotted again. Thank you. 

**10. Discussion- Para 4, First sentence implies that tests are the only answer to the diagnosis of TBM, what about clinical prediction rules relying on clinical data- although so far they have not been very successful, neither have tests so a robust CPR may negate the need for tests.**

 I agree there is seldom a perfect test and clinical prediction rules can be extremely useful in areas where access to tests is limited. I look forward to seeing a CPR that can accurately distinguish TBM from other HIV-related brain infections / pathology and commend researchers in this pursuit. 

**Discussion- Para5, Do the authors really think there are dead bacilli in CSF? Some of the authors have argued the opposite in their recent paper on Xpert Ultra so you can’t have it both ways.**

 The presence of MTB DNA in the CSF of a immunocompromised adult with clinical meningitis in a TB endemic setting almost certainly represents TBM. However it may not always be possible to culture organisms as they may have been rendered non-viable by recent TB treatment, inflammatory response or delays in processing. 

 ​​​​​​​Thanks again for your comments. We hope the revised manuscript is to your satisfaction. 
